# Flow Cytometry-Based Detection of Minimal/Measurable Residual Disease Predicts Survival Outcomes in Pediatrics, Adolescents, and Young Adults With T-acute Lymphoblastic Leukemia

**DOI:** 10.7759/cureus.61705

**Published:** 2024-06-05

**Authors:** Priyavadhana Balasubramanian, Jay Singh, Amar Ranjan, Pranay Tanwar, Sameer Bakhshi, Anita Chopra

**Affiliations:** 1 Pathology and Laboratory Medicine, All India Institute of Medical Sciences Rishikesh, Rishikesh, IND; 2 Laboratory Oncology, Institute Rotary Cancer Hospital, All India Institute of Medical Sciences, New Delhi, New Delhi, IND; 3 Medical Oncology, All India Institute of Medical Sciences, New Delhi, New Delhi, IND

**Keywords:** minimal/measurable residual disease, flow cytometry, t-acute lymphoblastic leukemia, adolescents and young adults, pediatric

## Abstract

Background: Measurable/minimal residual disease (MRD) is considered the single most powerful high-risk factor in acute leukemia, including T-cell acute lymphoblastic leukemia (T-ALL). In this study, we evaluated the impact of flow cytometry (FC)-based detection of MRD on survival outcomes in pediatrics, adolescents, and young adults (AYA) with T-ALL.

Methods: We included 139 patients, 88 pediatric patients between the ages of one and 14 years, and 51 AYA patients between 15 and 39 years of age, over a period of three years and were treated with the Indian Collaborative Childhood Leukemia Group (ICiCLe) protocol. MRD assessment was performed on post-induction (PI) bone marrow aspirate samples using a 10-color 11-antibody MRD panel on a Gallios instrument (Beckman Coulter, Miami, FL, USA). MRD value > 0.01% was considered positive. PI-MRD status was available in 131 patients.

Results: The five-year event-free survival (5-year EFS) in PI-MRD positive patients was inferior to those of negative patients (13.56% vs 79.06%), which was statistically significant (P < 0.001). However, the five-year overall survival (5-year OS) did not show any statistically significant difference between PI-MRD positive and negative T-ALL patients (92.93% vs 94.28%). The hazard ratio (HR) for 5-year EFS and MRD positivity was 8.03 (p-value < 0.0001). HR for 5-year EFS and early T-cell precursor ALL (ETP-ALL) was 2.63 (p = -0.02).

Conclusions: PI-MRD detected using FC is a strong predictive factor of inferior survival outcomes in pediatrics, AYA patients with T-ALL. PI-MRD positivity can be used to modify the treatment of T-ALL patients, especially in resource-constrained developing countries where molecular tests are not widely available.

## Introduction

Measurable/minimal residual disease (MRD) is considered the single most powerful high-risk factor in acute leukemias. Although there are many studies in the literature on MRD and survival outcomes in pediatric B-acute lymphoblastic leukemias [1-4], data on the same in T-cell acute lymphoblastic leukemias (T-ALLs) are scarce which are mostly from the Western population [5,6] and very few from developing countries [7]. Previous studies have shown that MRD positivity is associated with inferior survival outcomes in T-ALL. There is hardly any literature on adolescents and young adults (AYA) with T-ALL. MRD monitoring can be a reliable marker for risk stratification and management of patients, especially in low- and middle-income countries (LMIC).

MRD can be monitored using either of two methods: multicolor flow cytometry-based MRD (MFC-MRD) or quantitative polymerase chain reaction-based MRD (PCR-MRD). MFC-MRD has the advantages of easy processing, relatively inexpensive, wider applicability, and faster results whereas PCR-MRD is time-consuming, labor-intensive, expensive, and needs extensive standardization [6,8-11]. In this study, we evaluated the impact of flow cytometry (FC)-based MRD detection on survival outcomes in pediatrics and AYA with T-ALL from a tertiary cancer hospital in India.

## Materials and methods

This study was conducted on 139 newly diagnosed T-ALL patients, 88 pediatric T-ALL patients (age <15 years), and 51 AYA with T-ALL (age 15-39 years), treated with Indian Collaborative Childhood Leukemia Group (ICiCLe) protocol. The study was approved by the Institute Ethics Committee. Patients who were treated in other hospitals, those who refused to continue treatment in our center, and ≥5% MFC-MRD post-induction (PI) were excluded. In all cases, T-ALL diagnosis was based on morphology, cytochemistry (Myeloperoxidase and Periodic acid Schiff), and flow cytometric immunophenotyping.

All the patients were treated with the ICiCLe protocol, which is a prospective, randomized, open-label, collaborative, multicentric, risk-stratified, controlled therapeutic trial for newly diagnosed childhood ALL in India. Briefly, the ICiCLe protocol consisted of five phases: induction, consolidation, interim maintenance, delayed intensification, and maintenance. The induction phase was for 35 days (weeks 1-5) consisting of prednisolone, dexamethasone, vincristine, intrathecal methotrexate (IT MTx), asparaginase, and daunorubicin. The consolidation phase was for 63 days (weeks 6-14) consisting of IT MTx, cyclophosphamide, asparaginase, cytarabine, vincristine, and 6-Mercaptopurine (6-MP). Interim maintenance was for 56 days (weeks 11-18) consisting of IT MTx, intravenous methotrexate, folinic acid, and 6-MP. Delayed intensification was for 49 days (weeks 23-29) consisting of pulsed dexamethasone, vincristine, IT MTx, asparaginase, cyclophosphamide, cytarabine, 6-MP, doxorubicin, and mitoxantrone. The maintenance phase was for 84 days (eight cycles, 12 weeks each) consisting of oral MTx and 6-MP. CNS-directed therapy consisted of IT MTx at various stages of treatment [12].

MFC-MRD monitoring

MFC-MRD was performed in bone marrow aspirate (BMA) samples at the end of induction (PI days 35-40). BMA samples were processed by the bulk-lysis protocol. Briefly, an ammonium chloride-based lysing solution was used to bulk lyse the red blood cells, and the cells were stained with the 11 antibody MRD panel [13].

The primary MFC-MRD panel consisted of the following antibodies: surface CD3, intracellular CD3, CD4, CD5, CD7, CD8, CD16, CD34, CD38, CD45, and CD56. We also used an additional panel of antibodies comprising CD1a, CD2, CD11b, CD13, CD33, CD117and TdT in samples where the primary panel was of less help [7].

The cells were acquired on the Gallios flow cytometer (Beckman Coulter, Miami, FL, USA). Instrument set-up and daily quality controls were done per the company’s recommendations. The limit of detection (LOD) for MRD assay was established at a cluster of 20 events in 1.5 million cells (i.e., 0.0013%) and a lower limit of quantitation (LOQ) at a cluster of 50 events in 1.5 million cells (i.e., 0.003%). We could acquire 1-1.5 million cells in all MRD samples. FC data analysis was performed using Kaluza-software version 1.3 (Beckman Coulter, Miami, USA) [7].

Statistical analysis

Statistical analysis was performed using Stata (StataCorp, College Station, TX) and SPSS software (IBM Corp., Armonk, NY). Fisher’s exact test was used for the association between baseline characteristics and PI-MRD. Overall survival (OS) was taken from the date of initiation of chemotherapy until the date of death. Event-free survival (EFS) was determined from the date of initiation of chemotherapy until the date of any event such as relapse (medullary or isolated CNS or testicular) or relapse-free death [7].

The association of factors, such as age, gender, total leucocyte counts at diagnosis, early T-cell precursor ALL (ETP-ALL), or PI-MRD status, with OS and EFS was studied using the Kaplan-Meier method. Multivariate analysis was performed using the Cox proportional Hazards model. P-value < 0.05 was considered statistically significant.

## Results

Patients and samples

We studied a total of 139 patients, 88 children and 51 AYA, diagnosed as T-ALL and treated with ICiCLe protocol. The median age at diagnosis in children was 9.5 years (range 1-14 years) whereas in AYA patients, the median age was 20 years (range 15-38 years). The male-to-female ratio was 7:1 in the pediatric age group and 11.8:1 in AYA patients. The median WBC count at diagnosis was 31.6 × 10^9^/L in pediatric T-ALL and 24.36 × 10^9^/L in AYA T-ALL patients. Hyperleukocytosis defined as WBC counts ≥100 × 10^9^/L at diagnosis, was found in 30.7% of pediatric cases and 19.6% of AYA patients. Bone marrow was in morphological remission in 83 (94%) pediatric patients and 46 (90%) AYA patients.

There were only five (5.7%) ETP-ALL patients in the pediatric age group compared to 10 (19.6%) patients in AYA. Pediatric patients were immunophenotypically subclassified as pro-T-ALL (13, 15.77%), pre-T-ALL (16, 19.3%), cortical T-ALL (32, 38.5%), and mature T-ALL (22, 26.5%) according to European Group for the Immunological Classification of Leukemias (EGIL). Among the AYA patients, cortical T-ALL was the most common (17, 41.5%) and pro-T-ALL, pre-T-ALL, and mature T-ALL were equally distributed (eight, 19.5%). The characteristics of pediatric and AYA T-ALL are mentioned in Table 1.

**Table 1:** Characteristics of pediatric and AYA T-cell lymphoblastic leukemias NCI - National Cancer Institute, MRD - Minimal/Measurable Residual Disease, ETP - Early T-cell precursor, EGIL - European Group for the Immunological Classification of Leukemias.

	Pediatric		Adult	
Parameters	Median	Range	Median	Range
Age (in years)	9.5	01-14	20	15-38
Hemoglobin (g/dL)	9	3.6-14.3	9.85	4.5-18.4
Total Leucocyte counts 10^9^ /liter	31.6	1.04-796	24.36	0.580-663
Platelet count 10^9^/liter	37	5-1060	63	9-350
Blasts % at diagnosis				
Parameters	Number (N=88)	Frequency (%)	Number (51)	Frequency (%)
Males	77	87.5	47	92.2
Females	11	12.5	4	7.8
Male:female ratio	7		11.8	
NCI Risk				
Low	26	29.5	51	100
High	62	70.5	0	0
Morphological (Bone marrow) remission (MR)				
In MR	84	95.5	46	90.2
Not in MR	4	4.5	5	9.8
Post-induction MRD	N= 83		N=51	
Positive	18	21.6	12	23.5
Negative	65	78.3	39	76.5
ETP vs non-ETP				
ETP	5	94.3	10	80.4
Non-ETP	83	5.7	41	19.6
EGIL				
Pro	13	17	8	19.5
Pre	16	21.6	8	19.5
Cortical	32	36.4	17	41.5
Mature	22	25	8	19.5

MRD results

End-of-induction (EOI) MRD was available in 131 patients (83/88 pediatrics and 48/51 AYA patients). MRD positivity was noted in 18 (21.7%). among the pediatric population and in 12 (23.5%) patients among AYA patients.

Follow-up and outcome

**Figure 1:**
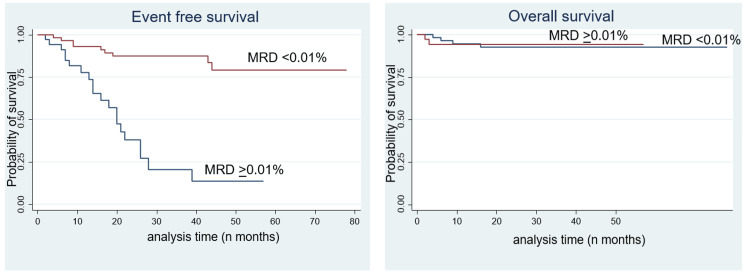
(Left) Event-free survival and (right) overall survival for patients stratified by PI-MRD status (Kaplan Meir analysis) PI - Post induction, MRD - Minimal/Measurable Residual disease

The median follow-up was 21.5 months. The prognostic value of MRD status with other risk factors, such as age, sex, and total leucocyte count, is given in Tables 2, 3. The five-year event-free survival (5-year EFS) in PI-MRD positive patients was inferior to that in MRD negative patients (13.56% vs 79.06%) which was statistically significant (p < 0.001). However, the five-year overall survival (5-year OS) did not show any statistically significant difference between PI-MRD positive and negative T-ALL patients (92.93% vs 94.28%) (Figure 1).

**Table 2:** Univariate analysis of MRD and other covariates with EFS and OS as the outcome TLC - Total leucocyte count, ETP - Early T-cell precursor, MRD - Minimal/Measurable Residual Disease

Variables	EFS			OS		
	HR	95% CI	P-value	HR	95% CI	P-value
Age (<10 vs >10 years	1.41	0.65-3.02	0.36	1.44	0.29-7.17	0.64
Sex (Male vs Female)	0.54	0.13-2.28	0.4	1.18	0.14-9.62	0.87
TLC (<50 x 10^9^/L vs >50 x 10^9^/L)	1.07	0.54-2.12	0.84	1.53	0.38-6.12	0.54
ETP-ALL vs non-ETP-ALL	2.63	1.12-6.15	0.02	2.26	0.45-11.2	0.31
Post-induction MRD (Positive vs Negative)	8.03	3.15-18.36	<0.0001	0.95	0.17-5.20	0.95

**Table 3:** Multivariate analysis of MRD and other covariates with EFS and OS as the outcome TLC - Total leucocyte count, ETP - Early T-cell precursor, MRD - Minimal/Measurable Residual Disease

Variables	EFS			OS		
	HR	95% CI	P-value	HR	95% CI	P-value
Age (<10 vs >10 years	1.27	0.40-3.62	0.73	2.74	0.27-27.09	0.38
Sex (Male vs Female)	0.45	0.05-3.88	0.47	3.73	0.37-37.10	0.26
TLC (<50 x 10^9^/L vs >50 x 10^9^/L)	1.27	0.54-2.97	0.57	0.87	0.15-4.93	0.88
ETP-ALL vs non-ETP-ALL	1.97	0.79-4.89	0.14	2.98	0.48-18.37	0.23
Post-induction MRD (Positive vs Negative)	6.03	2.39-15.16	<0.0001	0.79	0.11-4.28	0.70

The hazard ratio (HR) for 5-year EFS and MRD positivity was 8.03 with 95% CI 3.15-18.36 months with p-value < 0.0001. HR for 5-year EFS and ETP-ALL was 2.63 with 95% CI 1.12-6.15 with a p-value of 0.02. Other parameters were not statistically significant (Tables 2, 3).

Multivariate analysis showed statistically significant association of the EFS, and OS with MRD positivity status only. HR for 5-year OS and MRD positivity was 6.03 with 95% CI 2.39-15.16 months with p-value < 0.0001 (Table 3).

## Discussion

MRD serves as important prognostic information in T-ALL, which can influence treatment options in day-to-day practice [14,15]. However, in developing countries, other than MRD status, there are many host factors like malnutrition, poor socioeconomic status, increased rates of infections, limited treatment options, and drug toxicities, which might also have an important role in treatment decisions [16-19].

We are still unclear about the practical utility of MRD monitoring amid all these challenges. In this study, we assessed the predictive value of MRD monitoring in the management of pediatric and AYA T-ALL treated with ICiCLe protocol. Our data showed that PI-MRD-positive patients had a statistically significant higher risk of relapse. The 5-year EFS in PI-MRD-positive patients was inferior to MRD-negative patients (13.56% vs 79.06%).

As reported in other studies, this study also complements the prognostic value of PI-MRD in the treatment of T-ALL [6]. FCM-based MRD assessments have been demonstrated to serve as a reliable predictor for relapse in T-ALL [6,20].

Different studies have used differing time points for the assessment of MRD for prognostication. Studies assessing the MRD status at the end of induction and consolidation have highlighted the prognostic impact of MRD status at both these time points. AIEOP-BFM-ALL 2000 study concluded that MRD status at day 78 determines relapse risk in T-ALL [21]. However, studies done by the Children’s Oncology Group (COG), NOPHO groups, and others have shown that EOI-MRD is the most appropriate predictor of relapse [6,20]. In LMIC, routine bone marrow testing at different time points may be challenging due to increased costs. Hence, it is very important to decide the time points for MRD assessment, especially in LMIC.

We used the cut-off of PI-MRD ≥ 0.01% as concurrent with other studies [6,20] which had found that PI-MRD ≥ 0.01% had inferior outcomes. Hyperleukocytosis is considered a poor prognostic factor in ALL [22] and WBC count > 200,000/microliter is associated with inferior outcomes in non-ETP-ALL [20]. In this study, hyperleukocytosis was much higher in the pediatric population when compared to the AYA group (30.7% vs 19.6%, respectively). However, these frequencies are lower than those reported in the literature [7,22]. In our study, hyperleukocytosis did not show any statistically significant association with EFS and OS.

Similarly, the frequency of ETP-ALL in the pediatric age group was lower in the present study (5.7%) when compared with the literature (11% to 13%) [7,20]. ETP-ALL showed a statistically significant lower EFS compared to non-ETP-ALL in this study. However, the COG AALL0434 study found that both groups had similar outcomes. This could be due to better treatment facilities available in the developed countries [20].

Other characteristics like age and sex failed to show an independent association with poor EFS or OS which is in agreement with the report of Tembhare et al.'s COG study AALL0434 and the report of Conter et al. [7,20,23].

Overall, the outcomes of T-ALL are inferior in developing nations when compared to developed countries. There can be variable reasons for this lower survival outcome like patients presenting late to the hospital and infections, which might affect the predictive value of MRD. The limitation of this study is less sample size. We used the ICiCLe protocol, which is different from the protocols used in other studies [7,21], which might have also affected the outcome. However, taking these limitations into account, the results of this study clearly show a statistically significant difference between the MRD-positive and MRD-negative patients.

## Conclusions

To conclude, we report the prognostic impact of FC-based MRD in the management of T-ALL in pediatric and AYA patients from a referral cancer center treated with ICiCLe protocol. Despite the difficulties and challenges faced, PI-MRD is the single most relevant prognostic factor in T-ALL.
